# An improved method for detecting circulating microRNAs with S-Poly(T) Plus real-time PCR

**DOI:** 10.1038/srep15100

**Published:** 2015-10-13

**Authors:** Yanqin Niu, Limin Zhang, Huiling Qiu, Yike Wu, Zhiwei Wang, Yujia Zai, Lin Liu, Junle Qu, Kang Kang, Deming Gou

**Affiliations:** 1 Shenzhen Key Laboratory of Microbial Genetic Engineering, Shenzhen Key Laboratory of Marine Bioresource and Eco-environmental Science, College of Life Sciences, Shenzhen University, Shenzhen, Guangdong, 518060, China; 2 Key Laboratory of Optoelectronic Devices and Systems of Ministry of Education and Guangdong Province, College of Optoelectronic Engineering Shenzhen University, Shenzhen, Guangdong, 518060, China; 3 Department of Cardiovascular Surgery, Shenzhen Sun Yat-Sen Cardiovascular Hospital, Shenzhen, Guangdong, 518060, China; 4 Department of Physiological Sciences, Oklahoma State University, Stillwater, OK 74048, USA; 5 Department of Physiology, Shenzhen University Health Science Center, Shenzhen, Guangdong, 518000, China

## Abstract

We herein describe a simple, sensitive and specific method for analysis of circulating microRNAs (miRNA), termed S-Poly(T) Plus real-time PCR assay. This new method is based on our previously developed S-Poly(T) method, in which a unique S-Poly(T) primer is used during reverse-transcription to increase sensitivity and specificity. Further increased sensitivity and simplicity of S-Poly(T) Plus, in comparison with the S-Poly(T) method, were achieved by a single-step, multiple-stage reaction, where RNAs were polyadenylated and reverse-transcribed at the same time. The sensitivity of circulating miRNA detection was further improved by a modified method of total RNA isolation from serum/plasma, S/P miRsol, in which glycogen was used to increase the RNA yield. We validated our methods by quantifying miRNA expression profiles in the sera of the patients with pulmonary arterial hypertension associated with congenital heart disease. In conclusion, we developed a simple, sensitive, and specific method for detecting circulating miRNAs that allows the measurement of 266 miRNAs from 100 μl of serum or plasma. This method presents a promising tool for basic miRNA research and clinical diagnosis of human diseases based on miRNA biomarkers.

MicroRNAs (miRNAs) are small noncoding RNAs of ∼22 nucleotide (nt) in length. They exist in almost all eukaryotic organisms and regulate gene expression by binding the 3′-untranslated region (3′-UTR) of target mRNAs^1^,^2^,^3^. Once a miRNA binds to its target mRNA, protein translation is inhibited or the mRNA is degraded via the miRNA-mediated RNA interference^1^,^4^. miRNAs are involved in cell differentiation, proliferation, and apoptosis and are implicated in many types of diseases^5^,^6^,^7^,^8^,^9^. miRNA expression profiles differ between healthy and diseased tissue^10^,^11^,^12^. Furthermore, miRNAs circulate in blood in a surprisingly high stable form^13^. Thus, circulating miRNAs have the remarkable potential to be developed as diagnostic, prognostic, and predictive biomarkers^14^,^15^,^16^.

The quantitative real-time PCR (qRT-PCR) has been recognized as one of the most sensitive techniques for miRNA detection^17^,^18^,^19^ although other methods for quantifying miRNA are available, such as northern blotting, microarray or deep sequencing. Several approaches have been reported for detection of miRNAs using qRT-PCR. The most popular stem-loop method requires unique reverse transcription primers and a specific probe for each miRNA assay, and therefore it is fairly costly for screening of a large number of miRNAs^17^. The poly(A) method relies on adding a poly(A) tail to all types of RNAs, including miRNAs, and using a generic oligo(dT) primer for the reverse transcription (RT)^20^. This method is capable of assaying miRNA expression in a high-throughput manner. However, it is less specific due to the non-specific reverse transcription. We have previously developed a novel S-Poly(T) method for miRNA qPCR assay, which is based on a uniquely designed S-Poly(T) reverse-transcription (RT) primer that contains six miRNA-specific bases and a oligo(dT) sequence^21^. Although the sensitivity of the S-Poly(T) method is at least 4 times higher than that of the stem-loop and poly(A) methods, the S-Poly(T) method is still time-consuming and less-efficient since polyadenylation and reverse transcription are performed separately.

Therefore, there is a need for further optimization of miRNA quantitation using qRT-PCR, particularly for high-throughput screening or clinical samples with low miRNA levels, such as human serum/plasma (S/P). Based on our recently developed S-Poly(T) method^21^, here we describe an improved method for detecting circulating miRNAs, termed S-Poly(T) Plus. The novelty of this approach is the combination of polyadenylation and reverse transcription into one step with retaining the S-Poly(T) primer. We optimized the experimental protocol for the S-Poly(T) Plus method. We also validated this method by quantifying miRNA expression profiles in the sera of pulmonary arterial hypertension (PAH) associated with congenital heart disease (CHD-PAH) patients. We finally highlighted the strengths and limitations of this approach for clinical applications.

## Results

### Design of S-Poly(T) Plus method

**Figure 1:**
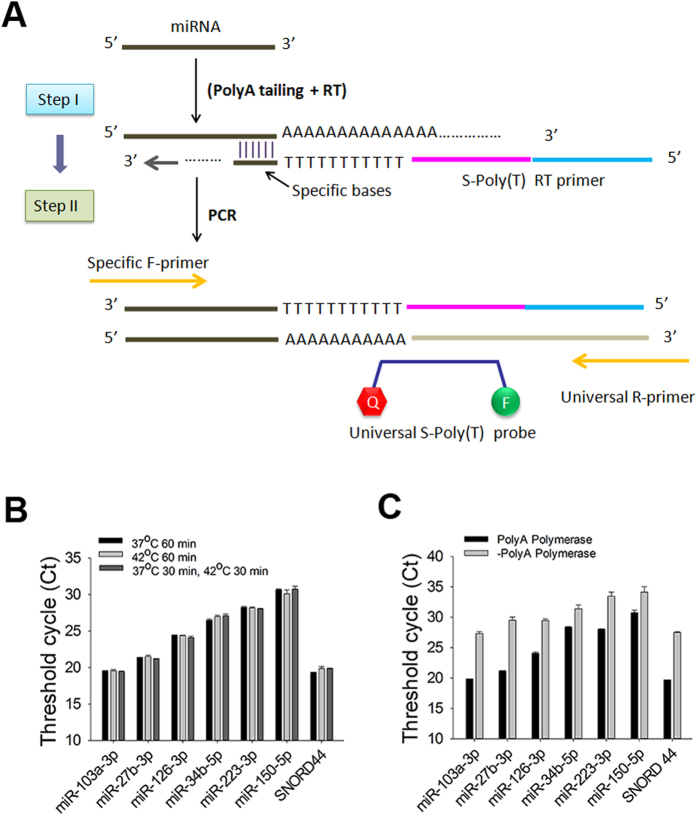
Optimization of S-Poly(T) Plus method for circulating miRNA assay. (**A**) A schematic representation of the S-Poly(T) Plus method for miRNA quantification. miRNAs are polyadenylated and reverse-transcribed into cDNA simultaneously. The S-Poly(T) RT primer consists of four segments, in 5′ to 3′ direction, a universal reverse primer sequence, a universal Taqman probe sequence and 17 bases (11 dT and 6 specific bases) that are complementary to the 3′ end of a particular miRNA with tailed poly(A). The PCR products are generated with a miRNA-specific forward primer and a universal reverse primer and detected by a universal Taqman probe. (**B**) Effect of reaction temperatures on polyA polymerase and reverse transcriptase. (**C**) Effects of polyA polymerase on the detection of miRNAs in the one-step polyadenylation/RT procedure. The expression levels of miR-103a-3p, miR-27b-3p, miR-126-3p, miR-34b-5p, miR-223-3p, miR-150-5p and SNORD44 in (**B**,**C**) were determined using total RNAs isolated from human HEK293A cells.

The higher sensitivity and specificity of S-Poly(T) method are achieved by the application of the S-Poly(T) reverse-transcription (RT) primer^21^. However, polyadenylation and reverse transcription in the S-Poly(T) method are carried out separately, which is time-consuming and costly. In the new version of S-Ploy(T) Plus method, we combined polyadenylation and reverse transcription into a one-step, multiple-stage reaction, which increased efficiency. With the optimized reaction buffer, the reaction time was reduced to 65 min compared to 105 min in the two-step reaction of the S-Poly(T) method; The RNA amount from serum/plasma needed for the assay was decreased to one-fifth (see the Methods). The schematic diagram of the S-Poly(T) Plus protocol was depicted in the Fig. 1A. All the primers and probes used in this study are listed in Supplemental file 1.

Because PolyA polymerase and reverse transcriptase could work at 37 ^o^C or 42 ^o^C, we tested the effects of temperatures on the efficiency of one-step, multiple-stage reaction. As shown in Fig. 1B, we did not observe significant differences in human HEK293A cells for all the miRNAs we tested under the three temperature conditions. We recommend using the condition of 37  ^o^C for 30 min and then 42  ^o^C for 30 min to retain the respective optimal reaction temperature of both PolyA polymerase and reverse transcriptase.

We also used human HEK293A cells to test whether Poly(A) polymerase is required for the one-step, multiple-stage reaction. The Ct values of miRNA qRT-PCR assays decreased by 3.0 ~ 8.3 units when Poly(A) polymerase was neglected in the reaction mixture (Fig. 1C), indicating that the polyadenylation step is critical for the improvement of sensitivity in S-Poly(T) Plus method.

### Sensitivity and dynamic range of S-Poly(T) Plus

**Figure 2:**
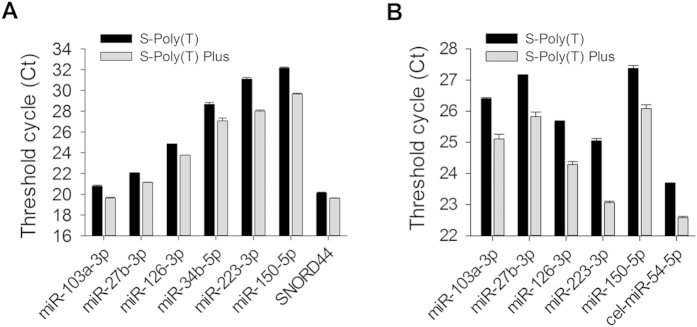
Comparison of sensitivity in miRNA assay between S-Poly(T) and S-Poly(T) Plus methods. (**A**) The expression levels of miR-103a-3p, miR-27b-3p, miR-126-3p, miR-34b-5p, miR-223-3p, miR-150-5p and SNORD44 were determined using total RNAs isolated from human HEK293A cells. (**B**) The expression levels of five human miRNAs of miR-103a-3p, miR-27b-3p, miR-126-3p, miR-223-3p, miR-150-5p and a spiked-in *Caenorhabditis elegans* miRNA cel-miR-54-5p were measured using total RNAs prepared from 24 healthy human serum mixture.

To demonstrate the improvement of the new method, the sensitivity between S-Poly(T) and S-Poly(T) Plus was compared. We first determined the expression levels of six miRNAs (miR-103a-3p, miR-27b-3p, miR-126-3p, miR-34b-5p, miR-223-3p, and miR-150-5p) and a snoRNA (SNORD44) in human HEK293A cells. The results showed that the Ct values of all the miRNAs as assayed by S-Poly(T) Plus were smaller than those by S-Poly(T) method. miR-223-3p showed the largest reduction in Ct value (3.07) and SNORD44 the smallest reduction (0.52) between two methods. Thus, S-Poly(T) Plus was 1.4 ~ 8.4-fold more sensitive than S-Poly(T) as calculated by 2^^-ΔCt^ method (Fig. 2A).

We also tested the sensitivity of S-Poly(T) Plus using human serum samples. As shown in Fig. 2B, all the Ct values of six human miRNAs (miR-103a-3p, miR-27b-3p, miR-126-3p, miR-223-3p, and miR-150-5p) and a spiked-in *Caenorhabditis elegans* cel-miR-54-5p were significantly reduced using S-Poly(T) Plus when compared to those using S-Poly(T) method. Similar to HEK293A cells, miR-223-3p displayed the largest difference in Ct value (2.00), and miR-27b-3p the smallest (1.11), with 2.2 ~ 4.0-fold differences in sensitivity between S-Poly(T) and S-Poly(T) Plus methods.

**Figure 3:**
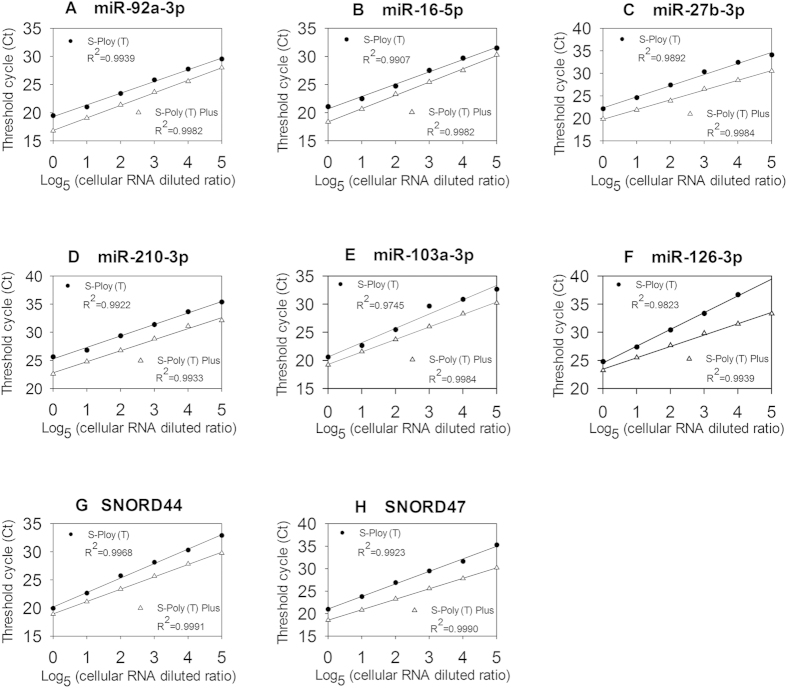
The correlation between total RNA inputs and threshold cycle (Ct) values of HEK293A cells as determined by S-Poly (T) and S-Poly (T) Plus methods. Total RNAs were isolated from human HEK293A cells. The RNA inputs ranged from 2.5 ng (original) to 0.8 pg (diluted 5^5^ times) for each real-time PCR reaction. (**A**) miR-92a-3p, (**B**) miR-16-5p, (**C**) miR-27b-3p, (**D**) miR-210-3p, (**E**) miR-103a-3p, (**F**) miR-126-3p, (**G**) SNORD44 and (H) SNORD47.

We further evaluated the dynamic range of S-Poly(T) Plus. Six miRNAs (miR-92a-3p, miR-16-5p, miR-27b-3p, miR-210-3p, miR-103a-3p and miR-126-3p) and two snoRNAs (SNORD44 and SNORD47) were selected for this purpose. Total RNAs from HEK293A cells ranged from 2.5 ng (original) to 0.8 pg (diluted 5^5^ times) were used for each miRNA detection. As shown in Fig. 3, the correlation coefficients (R^2^) produced by S-Poly(T) Plus and S-Poly(T) methods were 0.9933 ~ 0.9991 and 0.9745 ~ 0.9968, respectively. However, the regression lines of S-Poly(T) Plus were below those of S-Poly(T) method and there were 1.0 ~ 5.2 units difference in Ct value between two methods, indicating the increased sensitivity of S-Poly(T) Plus.

**Figure 4:**
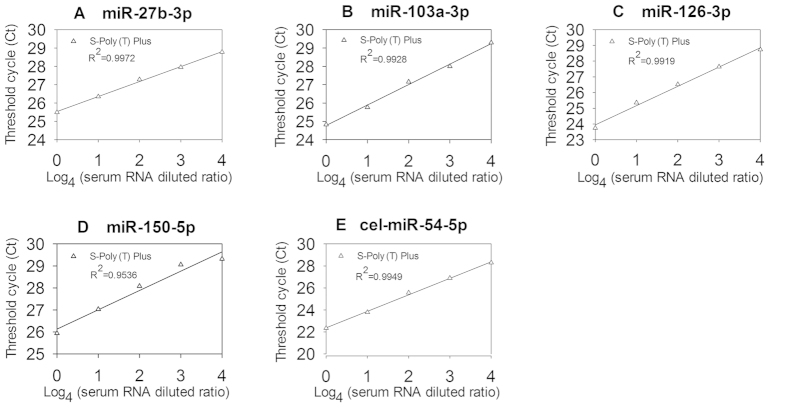
The correlation between total RNA inputs and threshold cycle (Ct) values of human sera as determined by S-Poly (T) Plus method. A 0.075 μl (original) ~ 0.3 nl (diluted 4^4^ times) of initial serum RNA, corresponding to 0.38 μl ~ 1.5 nl of human serum sample were used in each real-time PCR reaction. (**A**) miR-27-3p, (**B**) miR-103a-3p, (**C**) miR-126-3p, (**D**) miR-150-5p and (**E**) cel-miR-54-5p.

Human serum samples were further used to evaluate the dynamic range of S-Poly(T) Plus. Four miRNAs (miR-27-3p, miR-103a-3p, miR-126-3p and miR-150-5p) and a spiked-in cel-miR-54-5p were tested. We observed an excellent linear relationship between total RNA inputs and Ct values with R^2^ ranging between 0.9536 and 0.9972. Typically, 0.075 μl (original) ~ 0.3 nl (diluted 4^4^ times) of initial serum RNA, corresponding to 0.38 μl ~ 1.5 nl of human serum sample, was sufficient for detection of certain miRNA using S-Poly(T) Plus (Fig. 4).

### Optimization of RNA isolation

**Figure 5:**
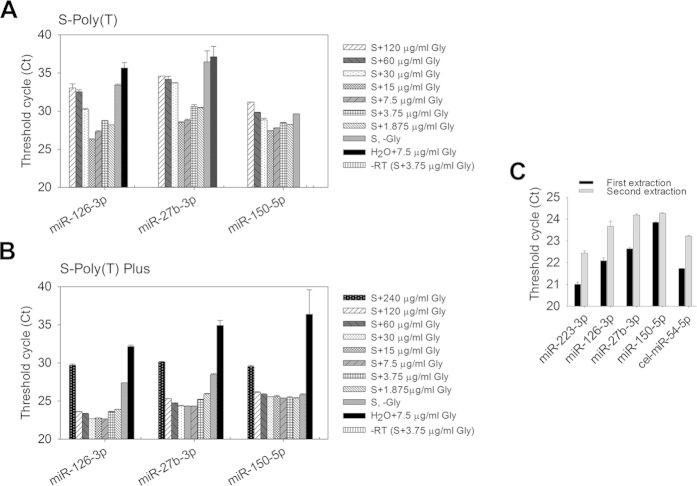
Optimization of serum RNA extraction using the co-precipitant, glycogen. Various concentrations of glycogen were applied to enhance the precipitation of serum RNA, which were used for detection of miRNAs using S-Poly(T) (**A**) and S-Poly(T) Plus (**B**). S: serum, Gly: glycogen and -RT: without reverse transcriptase. The concentrations of glycogen were 240 ~ 1.875 μg/ml. (**C**) Serum was subjected to the first or second extraction (see Method section for definitions for first/second extraction). The isolated RNA was assayed for miRNA levels using S-Poly(T) Plus method.

For circulating miRNA assay, total RNAs isolation from serum/plasma samples is very critical. To increase the yield of RNA isolation from serum or plasma samples, various concentrations of glycogen were added during the precipitation step of RNA extraction. We found that the optimized concentrations of glycogen were 1.875 ~ 15 μg/ml for S-Poly(T) method and 1.875 ~ 120 μg/ml for S-Poly(T) Plus (Fig. 5A,B). There was no significant signal detected in the controls without reverse transcriptase (-RT) using both S-Poly(T) and S-Poly(T) Plus methods. The smaller Ct values and a wider concentration range of glycogen suitable for RNA precipitation proved that the S-Poly(T) Plus assay was more sensitive and stable than the S-Poly(T) method. We routinely use 15 μg/ml of glycogen for RNA extraction for both S-Poly(T) and S-Poly(T) Plus methods and we named our RNA extraction method for serum/plasma samples S/P miRsol.

A previous study reported that an additional extraction of organic layer of serum samples can increase RNA yield when the amounts of samples are limited^22^. After the first extraction of RNAs as described above, we added 500 μl of water to the organic phase and collected the aqueous phase as the second extraction of RNAs after mixing and centrifugation. Using S-Poly(T) Plus method, we detected significant signals of the second extracted RNAs in miRNA qRT-PCR assay although the Ct values were 0.42 ~ 1.58 units larger than those of the first-extracted RNAs (Fig. 5C).

**Figure 6:**
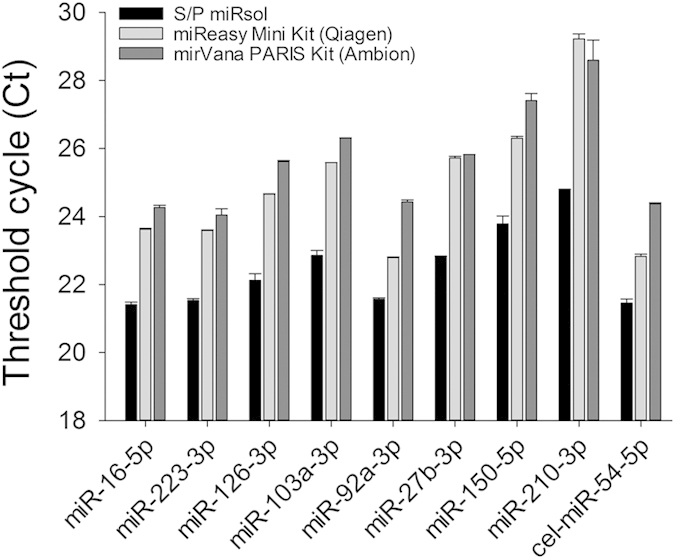
Comparison of the RNA isolation method, S/P miRsol developed in the current study with two commercial RNA isolation kits, miReasy Mini Kit (Qiagen) and mirVana PARIS Kit (Ambion). The efficiency of RNA extraction among different methods was evaluated based on Ct values measured by qPCR using an equal volume of initial serum.

We next compared our S/P miRsol with two commercially available RNA isolation kits, miReasy Mini Kit (Qiagen) and mirVana PARIS Kit (Ambion). Due to interference of glycogen on the absorbance of RNA, the concentration of total RNAs cannot be measured correctly by spectrophotometer. Therefore, we evaluated the efficiency of RNA extraction among different methods based on Ct values measured by qPCR using an equal volume of initial serum/plasma. The Ct values using S/P miRsol were decreased by 1.23 ~ 2.92 units compared to the miReasy Mini Kit and mirVana PARIS Kit, indicating that our method was more effective in serum RNA recovery for circulating miRNA assay (Fig. 6).

### microRNA expression profiling in CHD-PAH

**Figure 7:**
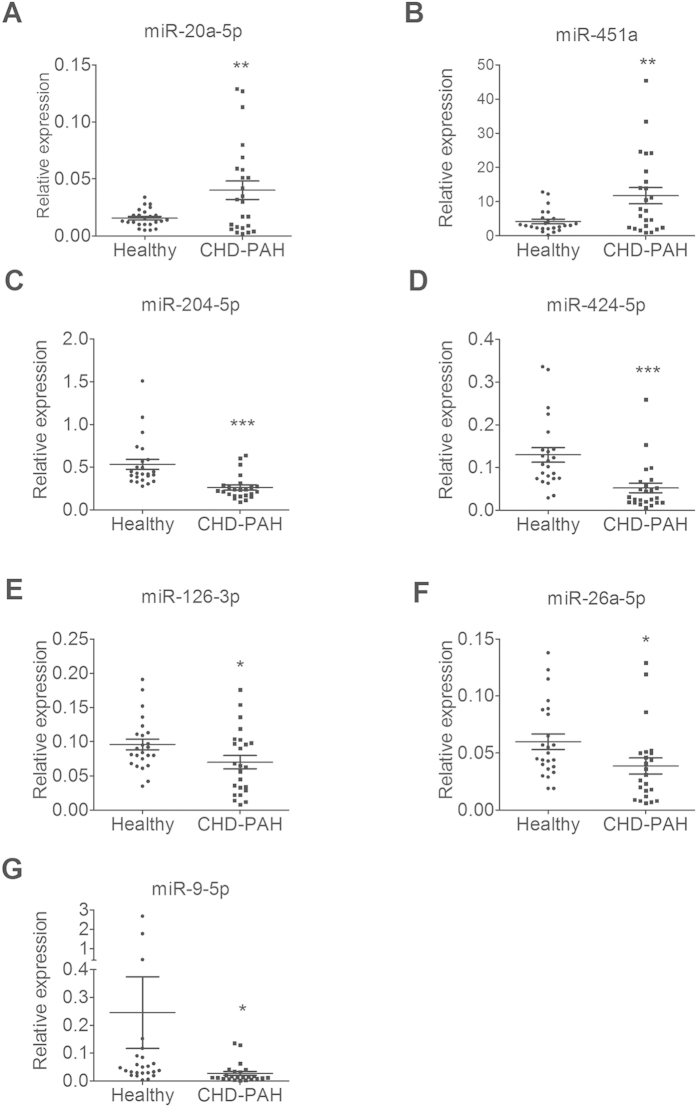
Differentially expressed miRNAs in the sera of CHD-PAH patients and healthy individuals. The expression levels of miRNAs were determined by S-Poly(T) Plus in the sera of 24 CHD-PAH patients and 24 healthy controls. miRNA levels were normalized to spiked-in cel-miR-54-5p and represented in scatter plots. Data are shown as means ± SE, *p < 0.1 v.s. healthy control, **p < 0.01 v.s. healthy control, ***p < 0.001 v.s. healthy controls.

miRNAs can be used as potential biomarkers for many human diseases. We tested the utility of S/P miRsol and S-Poly(T) Plus in clinical applications by determining miRNA profiles in sera. A total of 31 human miRNAs possibly related to pulmonary arterial hypertension (PAH) were selected based on previous reports (Supplemental file 2). The cel-miR-54-5p was used as a spiked-in normalization control. We first performed first screen by pooling serum samples of 24 healthy subjects and 24 patients with CHD-PAH and fourteen miRNAs (miR-451a, miR-9, miR-424, miR-223, miR-204, miR-150, miR-328, miR-21, miR-34a, miR-34b, miR-26a, miR-27b, miR-126 and miR-20a) changed more than 1.5-fold. These miRNAs were further validated in each of the sera from 24 CHD-PAH patients and 24 healthy subjects individually. We found that two miRNAs (miR-20a-5p and miR-451a) were significantly up-regulated and five miRNAs (miR-204-5p, miR-424-5p, miR-126-3p, miR-26a-5p and miR-9-5p) were significantly down-regulated in CHD-PAH sera compared to the healthy controls (Fig. 7).

**Figure 8:**
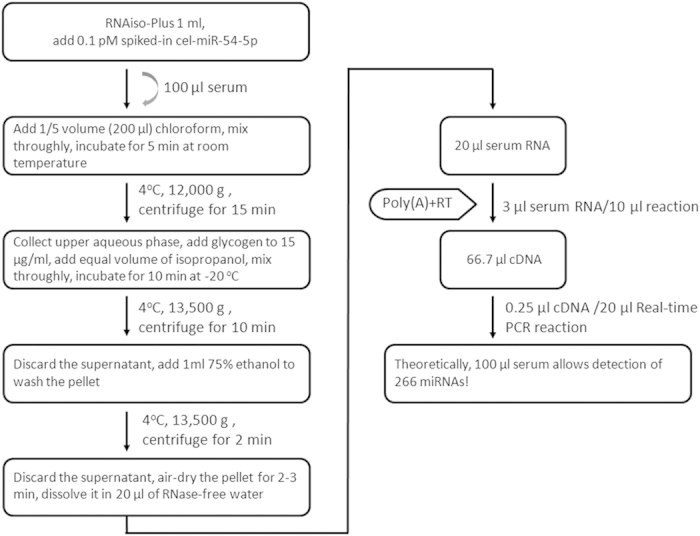
An optimized protocol for circulating miRNA analysis using S-Poly(T) Plus.

Taken all together, we developed an extremely sensitive and practical protocol for circulating miRNA detection by combining the S/P miRsol RNA extraction and the S-poly(T) Plus miRNA detection methods. If losses during the whole procedure are ignored, 100 μl of serum or plasma can be used to detect 266 miRNAs (Fig. 8).

## Discussion

The circulating miRNAs have the potential to be used as biomarkers in the diagnosis of human diseases^23^,^24^. In our previously reported S-Poly(T) method, a S-Poly(T) primer consisting of an oligo(dT)_11_ sequence and six miRNA-specific bases is used during reverse transcription, thereby providing higher binding strength and thermodynamic stability between RT primer and miRNA template. The sensitivity of S-Poly(T) assay was at least 4-fold higher than that of the widely used Stem-loop method^21^.

In the S-Poly(T) Plus assay reported in this study, RNAs were polyadenylated and reverse-transcribed with the S-Poly(T) primer in a single-step reaction. The sensitivity was increased by at least one fold compared to the S-Poly(T) method. The improved sensitivity could be due to the larger RNA substrate concentration in mixture reaction. In S-Poly(T) Plus method, 3 μl serum RNA was used in a 10 μl polyA tailing/RT reaction mixture. In S-Poly(T) method, 7.5 μl serum RNA was first used in a 10 μl of polyA tailing reaction mixture and only 2 μl of the polyadenylation reaction product containing 1.5 μl serum RNA was then used in a 10 μl of RT reaction mixture. Besides, less loss and lower risk of RNA degradation may also contribute to a higher sensitivity of S-Poly(T) Plus.

It is challenging to extract RNAs efficiently from serum/plasma because a tiny amount of RNAs is present in these samples. In this study, we successfully used the co-precipitant, glycogen to significantly improve the recovery of circulating miRNA (Figs 5 and 6). The S-Poly(T) Plus method is also cost-effective since all of the polyA-tailed RNA is used for RT reaction. By contrast, only one-fifth of polyadenylation product is added in the RT reaction in S-Poly(T) method. One hundred microliter of serum or plasma is sufficient for detecting 266 miRNAs using S-Poly(T) Plus method. Thus, the new method provides a powerful tool for miRNA screen when limited blood sample is available. For example, only limited amounts of blood can be drawn from transgenic mice; a droplet of blood can be used for point-of-care diagnosis of diseases.

PAH is characterized by vasculopathy due to increased vasoconstrictor tone and thrombosis. The structural changes are driven by excessive vascular cell growth and inflammation^25^. PAH occurs in different clinical conditions depending on associated diseases^26^, and it is commonly associated with congenital heart disease (15–30%)^27^. In the present study, the expression profiles of miRNAs in the sera of clinical CHD-PAH patients were validated by the S-Poly(T) Plus method. Our results revealed up-regulation of miR-20a-5p and miR-451a, and down-regulation of miR-204-5p, miR-424-5p, miR-126-3p, miR-26a-5p and miR-9-5p in the sera of CHD-PAH patients. These miRNAs may be of potential use as early detection biomarkers for CHD-PAH.

Reduced miR-150 levels have been observed in idiopathic PAH patients^23^. miR-210 is one of the validated miRNAs that are significantly induced by hypoxia in HPASMC, or other cell lines^21^,^28^. However, we did not observe any changes of these two miRNAs in the CHD-PAH patients. The discrepancies are not entirely unexpected and could be the results of the differential pathobiology induced by different diseases, drugs, or hypoxia^26^.

PAH is also characterized largely by enhanced proliferation and reduced apoptosis of PASMCs^29^. We have previously reported that miR-20a-5p directly targets the PRKG1 gene, thereby promoting the proliferation and migration of PASMCs but inhibiting cell differentiation^30^. We have also shown that miR-9-5p increases PAH-PASMC proliferation and resistance to apoptosis by enhancing the activity of NFAT via targeting KPNB1 and DYRK1B^31^. The down-regulation of miR-204 is found in PAH-PASMCs and pulmonary delivering of miR-204 reduces disease severity in a PAH animal model^32^. Other miRNAs among the 31 miRNAs selected for miRNA profiling in PAH have also been studied. For instance, the genetic ablation of miR-145 results in a significant decrease in PAH development in mouse models^33^,^34^. miR-451 promotes migration of PASMCs, but interestingly, the genetic loss of miR-451 does not attenuate the development of PAH. This is probably because the pathway redundancy compensates for the loss of miR-451^35^,^36^,^37^,^38^,^39^,^40^,^41^,^42^,^43^,^44^,^45^. In addition to PAH, cancer or other disease-specific miRNAs should further be explored for their clinical applications as reliable biomarkers.

In summary, the current study provides a simple, sensitive, and inexpensive miRNA profiling tool for detecting circulating miRNAs with the precise quantification. This tool could be used for diagnosis for a variety of human PAH, cancers or other diseases based on miRNA biomarkers.

## Methods

### Serum collection and cell culture

Human serum samples were collected from 24 healthy donors and 24 patients with CHD-PAH from the Yat-Sen Cardiovascular Hospital (Shenzhen, China). The blood samples were kept at room temperature for 1 h, and then centrifuged at 3,000 g for 10 min at 4 ^o^C. The sera were stored at −80 ^o^C. All procedures were carried out according to the approved guidelines. The Institutional Ethics Committee at the Sun Yat-Sen Cardiovascular Hospital (Shenzhen, China) approved the experiments. All participants were provided with written informed consent.

Human HEK293A cells were obtained from American Type Culture Collection (ATCC, Manassas, VA) and were maintained in Dulbecco’s modified Eagle’s medium (DMEM) supplemented with 10% fetal bovine serum (FBS) at 37 ^o^C and 5% CO_2_ condition.

### S/P miRsol RNA extraction

Circulating RNAs were isolated from serum/plasma by S/P miRsol method developed in this study. Briefly, 1 ml of RNAiso-Plus (TaKaRa, Dalian, China) supplemented with 0.1 pM spiked-in *Caenorhabditis elegans* cel-miR-54-5p for normalization was added to 100 μl of serum or plasma and quickly vortexed thoroughly. Then 200 μl of chloroform was added and mixed by vigorous shaking for 20 s. The samples were incubated for 5 min at room temperature and centrifuged at 12,000 g for 15 min at 4 ^o^C. A 500 μl of aqueous phase was transferred into a fresh tube and mixed with 6 μl of glycogen (AppliChem, Darmstadt, Germany). An equal volume (506 μl) of isopropyl alcohol was added into the mixture and incubated at −20 ^o^C for 10 min followed by centrifugation at 13,500 g for 10 min at 4 ^o^C. The RNA pellet was washed with 1 ml of 75% ethanol and dissolved in 20 μl of RNase-free water. The same procedure was used to isolate total RNAs from cultured cells except that no spiked-in cel-miR-54-5p and glycogen were used.

In certain experiments, second extraction was performed to see whether additional RNAs can be recovered from serum/plasma samples. Briefly, after 500 μl of aqueous phase was removed for the first exaction, an equal volume (500 μl) of RNase-free water was added to the organic phase. A 500 μl aqueous phase was collected as the second extraction of RNAs after mixing and centrifugation

### Polyadenylation and reverse transcription

For the S-Poly(T) method, total RNA from cultured cells or serum was polyadenylated with the Poly(A) Polymerase Tailing Kit (Epicentre, Madison, USA) according to manufacturer’s instruction. Briefly, 1 μl of 10 × poly(A) polymerase reaction buffer, 1 μl of 10 mM ATP and 0.8 units of Poly(A) polymerase were added to 500 ng total RNA from cells/tissues or 7.5 μl of 20 μl total RNAs isolated from 100 μl serum/plasma. The reaction mixture was made to a total volume of 10 μl with RNase-free water and incubated at 37 ^o^C for 30 min and then 65 ^o^C for 5 min. The tailed miRNA was reverse-transcribed in a 10 μl reaction containing 2 μl of polyadenylation reaction product, 1 μl of 0.5 μM RT primer, 2.5 μl of 2 mM dNTP, 1 μl of MMLV HP RT 10 × reaction buffer, and 100 units of MMLV High Performance Reverse Transcriptase (Epicentre, Madison, WY, USA). The reaction was incubated at 42 ^o^C for 60 min and terminated at 70 ^o^C for 10 min.

In the S-Poly(T) Plus method, polyadenylation and reverse transcription were performed in a single step with an optimized buffer. In brief, the following components were added to 10 μl of reaction mixture: 100 ng of total RNAs from cells/tissues or 3 μl of 20 μl total RNAs isolated from 100 μl of serum/plasma, 2.5 μl of 4 × reaction buffer mix containing ATP and dNTPs , 1 μl of 0.5 μM RT primer and 1 μl of polyA/RT enzyme mix (with 0.8 units of Poly(A) polymerase and 100 units of MMLV High Performance Reverse Transcriptase). The reaction was incubated at 37 ^o^C for 30 min, 42 ^o^C for 30 min, and then terminated at 75 ^o^C for 5 min.

To compare the sensitivity and dynamic range of two methods, the serially diluted RNAs were used in the polyadenylation and reverse transcription reaction. For RNA samples from cells, the amounts of RNAs for each qPCR reaction ranged from 2.5 ng (original) to 0.8 pg (diluted 5^5^ times). For serum RNA samples, the volumes of RNAs for each qPCR reaction ranged from 0.075 μl (original) to 0.3 nl (diluted 4^4^ times).

### Real-time PCR

For comparison, 0.5 μl cDNA for S-Poly(T) and 0.25 μl cDNA for S-Poly(T) Plus were used in real-time PCR reaction for serum/plasma samples to allow an equal amount of RNA inputs. An equal volume of cDNA (0.5 μl) was used in both methods for cell/tissue samples. The reaction mixture (20 μl) consisted of 4 μl 5 × qPCR Reaction Buffer, 0.5 unit hotstar Taq Polymerase (Geneup, Shenzhen, China), 0.4 μl 10 μM forward primer, 0.4 μl 10 μM universal reverse primer, 0.5 μl 10 μM universal Taqman probe, and 0.2 μl 100 × ROX reference dye. Real-time PCR was carried out on ABI StepOnePlus thermal cycler at the following conditions: 95 ^o^C for 3 min, followed by 40 cycles of 95 ^o^C for 10 s and 60 ^o^C for 30 s.

### miRNA profiling

A total of 31 human miRNAs possibly related to PAH were selected based on literatures and was listed in Supplemental file 2^36^,^37^,^38^,^39^,^40^,^41^,^42^,^43^,^44^,^45^. For the first screen, we pooled serum samples of 24 healthy subjects and 24 patients with CHD-PAH for miRNA profiling using the S-Poly(T) Plus method. Only those miRNAs with more than a 1.5-fold change in CHD-PAH group compared to healthy group in the first screen were selected for validation in each individual of 24 CHD-PAH patients and 24 healthy controls. Relative quantities of miRNAs were calculated using the 2^^-ΔCt^ method with cel-miR-54-5p spiked-in as a normalization control. Two-tailed Student’s test was used for statistical analysis using the GraphPad Prism 5. Data was shown as means ± SE (standard error).

## Additional Information

**How to cite this article**: Niu, Y. *et al.* An improved method for detecting circulating microRNAs with S-Poly(T) Plus real-time PCR. *Sci. Rep.* **5**, 15100; doi: 10.1038/srep15100 (2015).

## Supplementary Material

Supplementary Information
