# Supplementary material for: An improved method for detecting circulating microRNAs with S-Poly(T) Plus real-time PCR
Source: Sci Rep. 2015 Oct 13;5:15100. doi: 10.1038/srep15100 (PMC4602224; doi:10.1038/srep15100)
Supplement: Supplementary Information [file srep15100-s1.pdf]

An improved method for detecting circulating microRNAs with S-Poly(T) Plus real-time PCR

Yanqin Niu1,2*, Limin Zhang1*, Huiling Qiu1,2*, Yike Wu1, Zhiwei Wang3, Yujia Zai3, Lin Liu4, Junle Qu2, Kang Kang5#, Deming Gou1#

Supplemental file 1 Primers and probe sequences for S-Poly(T) Plus method.

miRNA	Sequence (5'-3')	Forward primer	RT primer
>hsa-miR-150-5p MIMAT0000451	UCUCCCAACCCUUGUACCAGUG	CCGGGTCTCCCAACCCTTGTA	GTGCAGGGTCCGAGGTCAGAGCCACCTGGGCAATTTTTTTTTTTCACTGG
>hsa-miR-23b-3p MIMAT0000418	AUCACAUUGCCAGGGAUUACC	TCGGATCACATTGCCAGGG	GTGCAGGGTCCGAGGTCAGAGCCACCTGGGCAATTTTTTTTTTTGGTAAT
>hsa-miR-130a-3p MIMAT0000425	CAGUGCAAUGUUAAAAGGGCAU	GTCGGCAGTGCAATGTTAAAA	GTGCAGGGTCCGAGGTCAGAGCCACCTGGGCAATTTTTTTTTTTATGCCC
>hsa-miR-191-5p MIMAT0000440	CAACGGAAUCCCAAAAGCAGCUG	CCGGGCAACGGAATCCCAAAAG	GTGCAGGGTCCGAGGTCAGAGCCACCTGGGCAATTTTTTTTTTTCAGCTG
>hsa-miR-30b-5p MIMAT0000420	UGUAAACAUCCUACACUCAGCU	TTCGGTGTAAACATCCTACAC	GTGCAGGGTCCGAGGTCAGAGCCACCTGGGCAATTTTTTTTTTTAGCTGA
>hsa-miR-133b MIMAT0000770	UUUGGUCCCCUUCAACCAGCUA	TCGGTTGGTCCCCTTCAAC	GTGCAGGGTCCGAGGTCAGAGCCACCTGGGCAATTTTTTTTTTTAGCTGG
>hsa-miR-208b-3p MIMAT0004960	AUAAGACGAACAAAAGGUUUGU	CCGGGATAAGACGAACAAAAG	GTGCAGGGTCCGAGGTCAGAGCCACCTGGGCAATTTTTTTTTTTACAAAC
>hsa-miR-1 MIMAT0000416	UGGAAUGUAAAGAAGUAUGUAU	CCGGGTGGAATGTAAAGAAGT	GTGCAGGGTCCGAGGTCAGAGCCACCTGGGCAATTTTTTTTTTTATACAT
>hsa-miR-26a-5p MIMAT0000082	UUCAAGUAAUCCAGGAUAGGCU	CCGGGTTCAAGTAATCCAGGA	GTGCAGGGTCCGAGGTCAGAGCCACCTGGGCAATTTTTTTTTTTAGCCTA
>hsa-miR-29c-3p MIMAT0000681	UAGCACCAUUUGAAAUCGGUUA	CCGGGTAGCACCATTTGAAAT	GTGCAGGGTCCGAGGTCAGAGCCACCTGGGCAATTTTTTTTTTTAACCGA
>hsa-miR-34b-3p MIMAT0004676	CAAUCACUAACUCCACUGCCAU	CCGGGCAATCACTAACTCCAC	GTGCAGGGTCCGAGGTCAGAGCCACCTGGGCAATTTTTTTTTTTATGGCA
>hsa-miR-451a MIMAT0001631	AAACCGUUACCAUUACUGAGUU	CCGGGAAACCGTTACCATTAC	GTGCAGGGTCCGAGGTCAGAGCCACCTGGGCAATTTTTTTTTTTAACTCA
>hsa-miR-1246 MIMAT0005898	AAUGGAUUUUUGGAGCAGG	CCGGGAATGGATTTTTGGA	GTGCAGGGTCCGAGGTCAGAGCCACCTGGGCAATTTTTTTTTTTCCTGCT
>hsa-miR-451a MIMAT0001631	AAACCGUUACCAUUACUGAGUU	CCGGGAAACCGTTACCATTAC	GTGCAGGGTCCGAGGTCAGAGCCACCTGGGCAATTTTTTTTTTTAACTCA
>hsa-miR-19a-3p MIMAT0000073	UGUGCAAAUCUAUGCAAAACUGA	TCGGTGTGCAAATCTATGCAA	GTGCAGGGTCCGAGGTCAGAGCCACCTGGGCAATTTTTTTTTTTCAGTTT
>hsa-miR-21-5p MIMAT0000076	UAGCUUAUCAGACUGAUGUUGA	TTCGGTAGCTTATCAGACTGA	GTGCAGGGTCCGAGGTCAGAGCCACCTGGGCAATTTTTTTTTTTCAACAT
>hsa-miR-204-5p MIMAT0000265	UUCCCUUUGUCAUCCUAUGCCU	TCGGTTCCCTTTGTCATCCT	GTGCAGGGTCCGAGGTCAGAGCCACCTGGGCAATTTTTTTTTTTAGGCAT
>hsa-miR-138-5p MIMAT0000430	AGCUGGUGUUGUGAAUCAGGCCG	CCGGGAGCTGGTGTTGTGAATC	GTGCAGGGTCCGAGGTCAGAGCCACCTGGGCAATTTTTTTTTTTCGGCCT
>hsa-miR-367-3p MIMAT0000719	AAUUGCACUUUAGCAAUGGUGA	CCGGGAATTGCACTTTAGCAA	GTGCAGGGTCCGAGGTCAGAGCCACCTGGGCAATTTTTTTTTTTCACCAT
>hsa-miR-27b-3p MIMAT0000419	UUCACAGUGGCUAAGUUCUGC	TTCGGTTCACAGTGGCTAAG	GTGCAGGGTCCGAGGTCAGAGCCACCTGGGCAATTTTTTTTTTTGCAGAA
>hsa-miR-302b-3p MIMAT0000715	UAAGUGCUUCCAUGUUUUAGUAG	TCGGTAAGTGCTTCCATGTTT	GTGCAGGGTCCGAGGTCAGAGCCACCTGGGCAATTTTTTTTTTTCTACTA
>hsa-miR-145-5p MIMAT0000437	GUCCAGUUUUCCCAGGAAUCCCU	CCGGGGTCCAGTTTTCCCAGGA	GTGCAGGGTCCGAGGTCAGAGCCACCTGGGCAATTTTTTTTTTTAGGGAT
>hsa-miR-20a-5p MIMAT0000075	UAAAGUGCUUAUAGUGCAGGUAG	TCGGTAAAGTGCTTATAGTGC	GTGCAGGGTCCGAGGTCAGAGCCACCTGGGCAATTTTTTTTTTTCTACCT
>hsa-miR-34a-5p MIMAT0000255	UGGCAGUGUCUUAGCUGGUUGU	CCGGGTGGCAGTGTCTTAGCT	GTGCAGGGTCCGAGGTCAGAGCCACCTGGGCAATTTTTTTTTTTACAACC
>hsa-miR-328-3p MIMAT0000752	CUGGCCCUCUCUGCCCUUCCGU	CCGGGCTGGCCCTCTCTGCCC	GTGCAGGGTCCGAGGTCAGAGCCACCTGGGCAATTTTTTTTTTTACGGAA
>hsa-miR-126-3p MIMAT0000445	UCGUACCGUGAGUAAUAAUGCG	GCGGGCGTACCGTGAGTAAT	GTGCAGGGTCCGAGGTCAGAGCCACCTGGGCAATTTTTTTTTTTCGCATT
>hsa-miR-424-5p MIMAT0001341	CAGCAGCAAUUCAUGUUUUGAA	GCCCGCCAGCAGCAATTCATGT	GTGCAGGGTCCGAGGTCAGAGCCACCTGGGCAATTTTTTTTTTTCAAAAC
>hsa-miR-503-5p MIMAT0002874	UAGCAGCGGGAACAGUUCUGCAG	CCGGGTAGCAGCGGGAACAGTT	GTGCAGGGTCCGAGGTCAGAGCCACCTGGGCAATTTTTTTTTTTCTGCAG
>hsa-miR-124-3p MIMAT0000422	UAAGGCACGCGGUGAAUGCC	CCGGGTAAGGCACGCGGTG	GTGCAGGGTCCGAGGTCAGAGCCACCTGGGCAATTTTTTTTTTTGGCATT
>hsa-miR-9-5p MIMAT0000441	UCUUUGGUUAUCUAGCUGUAUGA	TCGGTCTTTGGTTATCTAGCT	GTGCAGGGTCCGAGGTCAGAGCCACCTGGGCAATTTTTTTTTTTCATACA
>hsa-miR-223-3p MIMAT0000280	UGUCAGUUUGUCAAAUACCCCA	CACGGTGTCAGTTTGTCAAAT	GTGCAGGGTCCGAGGTCAGAGCCACCTGGGCAATTTTTTTTTTTGGGGTA
>hsa-miR-103a-3p MIMAT0000101	AGCAGCAUUGUACAGGGCUAUGA	GGAGCAGCATTGTACAGGG	GTGCAGGGTCCGAGGTCAGAGCCACCTGGGCAATTTTTTTTTTTCATAGC
>hsa-miR-92a-3p MIMAT0000092	UAUUGCACUUGUCCCGGCCUGU	TTCGGTATTGCACTTGTCCC	GTGCAGGGTCCGAGGTCAGAGCCACCTGGGCAATTTTTTTTTTTACAGGC
>hsa-miR-16-5p MIMAT0000069	UAGCAGCACGUAAAUAUUGGCG	TTCGGTAGCAGCACGTAAATA	GTGCAGGGTCCGAGGTCAGAGCCACCTGGGCAATTTTTTTTTTTCGCCAA
>hsa-miR-210-3p MIMAT0000267	CUGUGCGUGUGACAGCGGCUGA	CCGGGCTGTGCGTGTGACAGC	GTGCAGGGTCCGAGGTCAGAGCCACCTGGGCAATTTTTTTTTTTCAGCCG

miRNA	Sequence (5'-3')	Forward primer	RT primer
>cel-miR-54-5p MIMAT0020773	AGGAUAUGAGACGACGAGAACA	TCGGAGGATATGAGACGACG	GTGCAGGGTCCGAGGTCAGAGCCACCTGGGCAATTTTTTTTTTTGTTCTC
SNORD-44	CCTGGATGATGATAAGCAAATGCTGAC TGAACATGAAGGTCTTAATTAGCTCTAA CTGACT	TGGCCTGGATGATGATAAGCA	GTGCAGGGTCCGAGGTCAGAGCCACCTGGGCAATTTTTTTTTTTAGTCAG
SNORD-47	AACCAATGATGTAATGATTCTGCCAAAT GAAATATAATGATATCACTGTAAAACC GTTCCATTTTGATTCTGAGGTT	CGCCAATGATGTAATGATTCTG	GTGCAGGGTCCGAGGTCAGAGCCACCTGGGCAATTTTTTTTTTTAACCTC
Universal reverse primer	CAGTGCAGGGTCCGAGGT		
Universal Taqman probe	56-FAM/CAGAGCCAC/ZEN/CTGGGCAATTT/3IABkFQ		

Supplemental file 2 A list of 31 miRNAs possibly related to pulmonary arterial hypertension (PAH).

MicroRNA	Function	Up or down-regulated	Reference
miR-150	miR-150 levels correlate with 2-year survival in patients with PAH.	↓	
miR-23b, miR-130a, miR-191, miR-30b, miR-133b, miR-208b		↑	24
miR-1, miR-26a, miR-29c, miR-34b, miR-451a, miR-1246		↓	24
miR-451a		↑	35
miR-19a	miR-19a mediates the suppressive effect of laminar flow on cyclin D1 expression in human umbilical vein endothelial cells.		36
miR-21	Targets PPARα; increases the proliferation and migration of PASMC.	↑	37
miR-204	Targets SHP2; down-regulation of miR-204 activates NFAT. NFAT and SHP2 increase proliferation and resist apoptosis of PASMC.	↓	32
miR-138	Target Mst1; disrupt the Bcl-2 signaling pathway and negatively regulate PASMC apoptosis.		38
miR-367, miR-27b, miR-302b		↑	32
miR-145	Targets KLF5 gene and its downstream signaling molecule, myocardin.	↓	39
miR-20a	Targets the PRKG1; promotes the proliferation and migration of PASMC, but inhibits the differenation.	↑	30
miR-34a	The p53 gene deficiency in mice is associated with lower levels miR-34a and hypoxia-induced PH and vascular remodeling.	↓	40
miR-328	Inhibites L-type calcium channel-α1C suppresses the insulin growth factor 1 receptor, leading to apoptosis of pulmonary arterial smooth muscle cells.	↓	41
miR-126		↑	42
miR-424, miR-503	Targets FGF2 and FGFR1; induces the proliferation of PASMCs.	↓	43
miR-124	Inhibits NFAT pathway through targeting NFATc1 gene and two regulators, CAMTA1 and PTBP1.	↓	44
miR-9	Increased NFAT activation by targeting KPNB1 and DYRK1B.		31
miR-223	Contributes to PARP-1/STAT3 activation and thus to PAH development	↓	45
